# Electroacupuncture attenuates synovitis in knee osteoarthritis and is associated with modulation of the protein S-TAM (Axl/MerTK)-Rac1 signaling axis

**DOI:** 10.3389/fimmu.2026.1815290

**Published:** 2026-07-09

**Authors:** Meng-Meng Li, Fei-Yang Jia, Hua-Li Tang, Xing Wen, Xia-Rong Huang, Lin-Wei Yin, Yi-Yang Xiao, Meng-Jian Qu, Jun Zhou

**Affiliations:** 1 The First Affiliated Hospital of University of South China, Hengyang, China; 2 The First Affiliated Hospital, Department of Rehabilitation, Hengyang Medical School, University of South China, Hengyang, Hunan, China; 3 The Affiliated Nanhua Hospital, Department of Rehabilitation, Hengyang Medical School, University of South China, Hengyang, Hunan, China

**Keywords:** apoptotic cell clearance, electroacupuncture, knee osteoarthritis, protein S-TAM (Axl/MerTK)-Rac1 signaling axis, synovitis

## Abstract

**Background:**

Synovitis, a core pathological feature of knee osteoarthritis (KOA), drives pain and disease progression via sustained inflammation and disrupted tissue homeostasis. Electroacupuncture (EA) shows clinical benefits in KOA management, yet its specific molecular mechanisms against synovitis remain incompletely defined. The Protein S-Tyro3, Axl, MerTK (TAM) pathway—particularly Axl/MerTK and downstream Rac1—constitutes a key efferocytosis-related and inflammation-resolving signaling axis. We hypothesized that EA alleviates KOA synovitis and is associated with restoration of this dysregulated pathway.

**Methods:**

Male Sprague-Dawley rats were randomly assigned to Control, KOA (anterior cruciate ligament transection, ACLT), and KOA-EA groups. After 1 month of model induction, the KOA-EA group received EA at GB34, SP10, ST36, and KI3 (30 min/day, 5 days/week for 12 weeks; sparse-dense waves: 3/15 Hz, 1 mA). We assessed cartilage histopathology (Mankin’s/OARSI scores), synovitis (Krenn score), synovial apoptosis (TUNEL, Cleaved Caspase-3/F4/80 co-staining), serum cytokines (IL-1β, TNF-α, IL-10, TGF-β1 via ELISA), and MMP13 expression (IHC). qRT-PCR was used to measure Pros1, Axl, Mertk, and Rac1 mRNA expression in synovium, while Western blot was used to measure Protein S, Axl, MerTK, and Rac1 protein expression; MMP13 in cartilage was assessed by both methods.

**Results:**

ACLT successfully induced KOA, with severe cartilage degradation, synovial inflammation, elevated pro-inflammatory cytokines, and increased synovial apoptosis. EA significantly ameliorated cartilage damage (reduced Mankin’s/OARSI scores, *P* < 0.01), decreased MMP13 expression (*P* < 0.05), attenuated synovitis (lower Krenn score, *P* < 0.01), reduced synovial apoptosis (*P* < 0.001), and shifted the cytokine profile toward an anti-inflammatory pattern (reduced IL-1β/TNF-α and increased IL-10/TGF-β1, *P* < 0.05). EA was also associated with reversal of the KOA-induced downregulation of Protein S-TAM-Rac1 axis-related molecules, with significantly increased synovial mRNA expression and partial restoration of protein expression.

**Conclusion:**

EA showed anti-inflammatory and chondroprotective effects in this KOA model and was associated with changes in synovial Protein S-TAM (Axl/MerTK)-Rac1 axis-related molecules, with stronger evidence at the mRNA level than at the protein level. These molecular changes may be related to apoptotic cell clearance-related processes and inflammation resolution.

## 1 Introduction

Knee osteoarthritis (KOA) represents a leading cause of global disability, characterized by progressive degeneration of articular cartilage, subchondral bone remodeling, osteophyte formation, and—critically—synovial inflammation (synovitis) (1, 2). Once considered a mere consequence of cartilage “wear and tear,” synovitis is now recognized as an active driver of KOA pathogenesis, intimately linked to both pain perception and structural disease progression (3, 4). The inflamed synovium acts as a source of a catabolic and pro-inflammatory milieu, secreting cytokines such as interleukin-1β (IL-1β) and tumor necrosis factor-α (TNF-α), which stimulate chondrocytes and synovial fibroblasts to produce matrix-degrading enzymes like matrix metalloproteinase-13 (MMP13) and a disintegrin and metalloproteinase with thrombospondin motifs (ADAMTS), thereby accelerating cartilage breakdown (5–7). Furthermore, synovitis promotes angiogenesis and sensory nerve ingrowth, contributing directly to pain (8, 9). Consequently, therapies capable of mitigating synovitis hold significant promise not only for symptom relief but also for potentially modifying disease trajectory.

Current first-line pharmacological management for KOA, primarily consisting of oral non-steroidal anti-inflammatory drugs (NSAIDs) and intra-articular corticosteroid injections, focuses on symptomatic relief but is associated with well-documented systemic side effects and does not address the underlying dysfunctional tissue homeostasis (10, 11). This underscores the urgent need for safer, more holistic interventions that target the root inflammatory and reparative processes within the joint.

Electroacupuncture (EA), a modality that integrates traditional acupuncture with electrical stimulation, has emerged as a valuable complementary therapy for KOA. Accumulating clinical evidence from randomized controlled trials and meta-analyses supports its efficacy in reducing pain and improving physical function in KOA patients (12, 13). Preclinical studies have begun to unravel its mechanisms, suggesting roles in modulating inflammatory cytokine release, inhibiting pain pathway sensitization, and reducing cartilage catabolism (14, 15). However, a precise understanding of how EA specifically counteracts synovitis—a central pathological event—remains fragmented. Elucidating these mechanisms is essential for optimizing EA protocols and strengthening its integration into evidence-based practice.

A pivotal biological process that fails in chronic inflammatory conditions like KOA is the resolution of inflammation. Efficient resolution is not a passive cessation but an active program mediated by specialized pro-resolving mediators and cellular processes, chief among them being efferocytosis—the prompt phagocytic clearance of apoptotic cells (16, 17). When efferocytosis is impaired, apoptotic cells undergo secondary necrosis, releasing intracellular contents that perpetuate inflammation and tissue damage (18, 19). The Tyro3, Axl, and MerTK (TAM) family of receptor tyrosine kinases are master regulators of this resolution phase. Expressed on macrophages and other phagocytic cells, Axl and MerTK, upon engagement by their ligand Protein S, trigger intracellular signals that promote efferocytosis and simultaneously suppress pro-inflammatory nuclear factor-kappa B (NF-κB) signaling (20). A key downstream effector of MerTK-mediated engulfment is the small GTPase Rac1, which orchestrates the actin cytoskeletal rearrangements necessary for phagocytic cup formation (21). Thus, the Protein S-TAM (Axl/MerTK)-Rac1 axis constitutes a critical checkpoint for maintaining tissue homeostasis by ensuring the “silent” removal of apoptotic debris and dampening inflammation.

Interestingly, in chronic inflammatory arthropathies such as rheumatoid arthritis, this axis is often downregulated or dysfunctional, contributing to a state of “failed resolution” (22). Whether a similar deficit exists in KOA synovitis and, more importantly, whether it can be therapeutically targeted, is not yet known. Given EA’s documented immunomodulatory effects, we postulated that its benefits in KOA might involve the restoration of this pro-resolving pathway.

Therefore, this study aimed to investigate the protective effects of EA on synovitis and cartilage integrity in a rat model of KOA induced by anterior cruciate ligament transection (ACLT). We systematically evaluated histopathological changes, apoptosis, systemic inflammation, and the expression of catabolic and regulatory factors. Our central hypothesis was that EA attenuates KOA synovitis and is associated with changes in the Protein S-TAM (Axl/MerTK)-Rac1 signaling axis that may favor inflammation resolution and tissue repair. Clarifying this association may help to better understand the biological basis of EA in KOA.

## 2 Materials and methods

### 2.1 Experimental animals and grouping

Thirty 3-month-old specific pathogen-free (SPF) male Sprague-Dawley rats (weigh**t**ing 297 ± 12 g) were supplied by Hunan Slake Jingda Experimental Animal Co., Ltd. (License No.: SCXK [Xiang] 2020-0003). Animals were housed in a controlled environment with a temperature of 23 ± 2 °C, relative humidity of 50 ± 10%, and a 12-hour light/dark cycle. Standard rodent chow and water were provided ad libitum. After one week of acclimatization, rats were randomly divided into three groups using a computer-generated random number table: (1) Control Group (Control, n=10): Underwent sham surgery; (2) KOA Model Group (KOA, n=10): Underwent ACLT surgery to induce KOA; (3) EA Treatment Group (KOA-EA, n=10): Underwent ACLT surgery and received subsequent EA treatment. The sample size was determined based on previous similar studies and power analysis to ensure adequate statistical power. This animal study was approved by the Animal Ethics Committee of the University of South China (Approval No. USC2024DS026). All procedures were conducted in strict compliance with applicable national legislation and institutional requirements.

### 2.2 Main experimental reagents and instruments

Reagents: IL-1β(Proteintech, Batch No.: 40001895), TNF-α(Proteintech, Batch No.: 40001993), IL-10(Wuhan Huamei Biotech Co., Ltd., Batch No.: Q07038402), TGF-β1(Wuhan Huamei Biotech Co., Ltd., Batch No.: T08032210);Clearing Reagent for Histology(Servicebio, G1128);Hematoxylin-Eosin (HE) staining kit(Servicebio, G1076); Axl Antibody(Proteintech, Catalog No. 13196-1-AP);Racl Antibody (Abcam, Catalog No. ab155938);MerTk Antibody(Affinity, Catalog No. DF4785);Protein S Antibody(Bioss, Catalog No. bs-9512R);β-actin Antibody(Proteintech, Catalog No. 66009-1-Ig).

Instroments:Centrifuge(Hunan Xiangyi Laboratory Instrument Development Co., Ltd, H1650R);Multifunctional enzyme label instrument(Shenzhen Huisong Technology Development Co.,Ltd, MB-530);Slicer(Leica Microsystems Co., Ltd, RM2016);Microscope(Nikon Corporation, NIKON ECLIPSE E100);Image-forming system(Nikon Corporation, NIKON DS-U3);Electrophoresis Apparatus(Beijing Liuyi Biotechnology Co., Ltd, DYY-2C);Disposable Sterile Acupuncture Needles(Wujiang City Cloud & Dragon Medical Device Co., Ltd, 0.16×13mm);Hwato SDZ-IIB Electroacupuncture Instrument (Suzhou Medical Supplies Factory Co. Ltd);Transfer apparatus(Beijing Liuyi Biotechnology Co., Ltd, DYCZ-40D).

### 2.3 Model preparation (ACLT-induced KOA)

**Figure 1:**
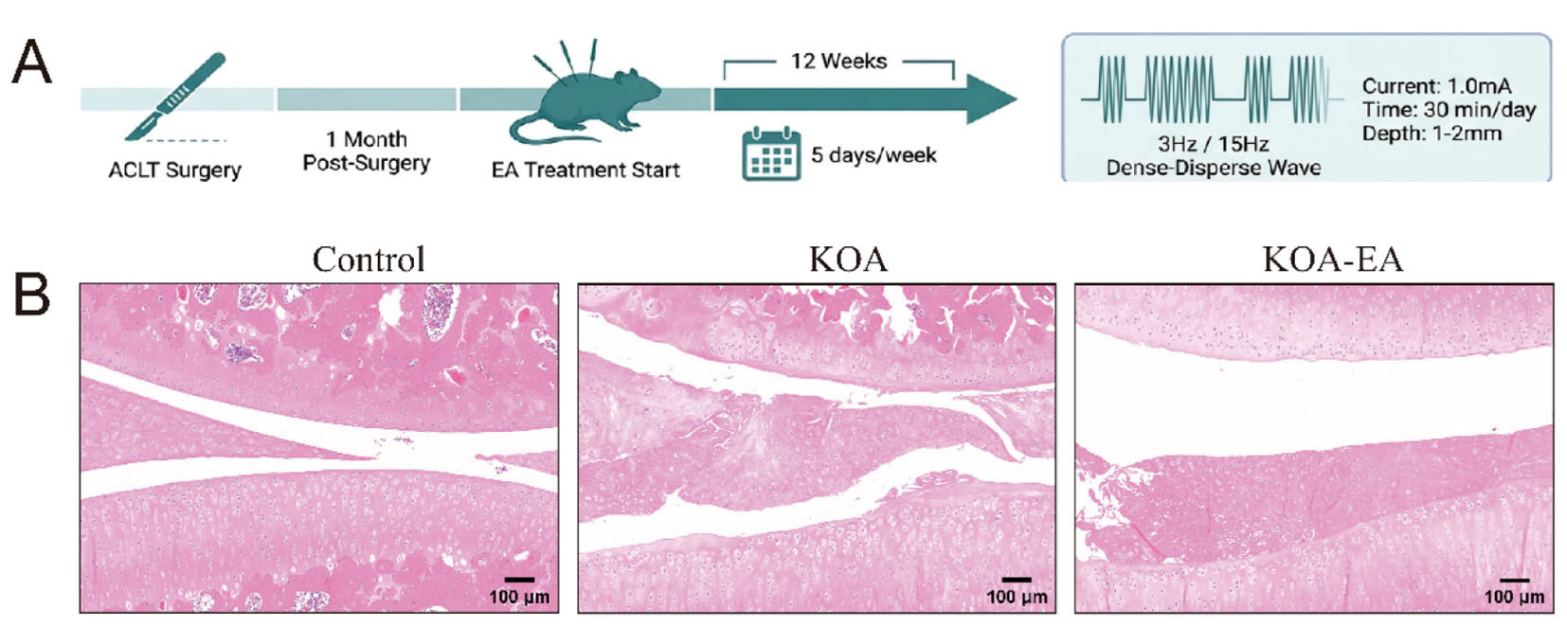
Schematic of the study. **(A)** Schematic diagram of the establishment of the ACLT-induced KOA model and EA intervention in rats. **(B)** KOA models were successfully induced, confirmed by HE staining of cartilage. H&E-stained sections were imaged at original magnification ×10, with scale bars of 100 μm. n = 2 rats per group.

Anterior cruciate ligament transection was performed to induce knee osteoarthritis (KOA) (23). Briefly, rats were anesthetized via an intraperitoneal injection of 1% sodium pentobarbital at a dose of 50 mg/kg. The anterior aspects of both knees were shaved and disinfected with iodine tincture. A medial parapatellar incision was made to expose the knee joint. The patella was dislocated laterally, and the anterior cruciate ligament (ACL) was visualized. The ACL was completely transected using micro-scissors. A positive anterior drawer test confirmed complete ACL rupture. The patella was reduced, and the joint capsule and skin were sutured layer by layer. The surgical area was disinfected again. Rats in the Control group underwent an identical surgical procedure including arthrotomy but without ACL transection. Postoperatively, all animals were allowed free movement in their cages. One month post−surgery, two rats from each group were randomly euthanized for preliminary histological assessment (H&E staining of the knee joint) to verify the successful establishment of the KOA model. These animals were used only for model verification and were excluded from the final statistical analyses. Successful modeling was confirmed in the KOA and KOA−EA groups when staining revealed characteristic pathological features, including cartilage surface fibrillation, chondrocyte clustering, proteoglycan loss, and synovial hyperplasia (**Figures 1A, B**).

### 2.4 Electroacupuncture intervention

EA treatment was initiated one month post-ACLT, which simulates a clinical intervention timeline following initial injury. Acupoint selection was based on traditional Chinese medicine theory for the treatment of “Bi Zheng” (arthralgia syndrome) and standardized animal atlases, specifically adhering to the guidelines outlined in “Name and Location of Commonly Used Acupoints in Experimental Animals – Part 2: Rat” (T/CAAM 0002–2020) (24). Bilateral acupoints were selected, including Yanglingquan (GB34), which is located on the lateral aspect of the hindlimb in the depression anterior and inferior to the head of the fibula, Xuehai (SP10), which is situated on the anteromedial aspect of the thigh over the prominence of the vastus medialis muscle, Zusanli (ST36), which lies lateral to the knee joint approximately 3 mm inferior to the fibular head, and Taixi (KI3), which is found in the depression midway between the medial malleolus and the Achilles tendon.

In the KOA-EA group, bilateral acupuncture was performed at GB34, SP10, ST36, and KI3 following skin disinfection. Sterile stainless steel needles (0.18 mm × 13 mm) were inserted to a depth of 1–2 mm, after which the needles were connected to an electrical acupuncture (EA) stimulator. A mixed sparse-dense wave (alternating frequencies of 3 Hz and 15 Hz) was applied with an intensity of 1.0 mA, adjusted to cause slight, visible local muscle twitching without eliciting distress or vocalization. EA stimulation lasted for 30 minutes per session, administered once daily, 5 days per week, for a continuous period of 12 weeks. Rats in the Control and KOA groups underwent identical handling and restraint procedures for an equivalent duration but received no needle insertion or electrical stimulation.

### 2.5 Sample collection

At the end of the 12-week treatment period, all rats were fasted overnight and then anesthetized via an intraperitoneal injection of 1% sodium pentobarbital at a dose of 50 mg/kg for deep anesthesia. Blood was collected from the abdominal aorta, centrifuged at 3000 rpm for 15 min, and the serum was aliquoted and stored at -80 °C for ELISA.

Rats were euthanized by cervical dislocation under deep anesthesia, and death was confirmed by cardiac and respiratory arrest. The intact right knee joints were harvested from each group of rats and fixed in 4% paraformaldehyde solution at room temperature for 48 consecutive hours. Subsequently, the fixed knee joints were subjected to decalcification, paraffin embedding, and serial sectioning, followed by histopathological analysis. Specifically, the histopathological analyses included hematoxylin and eosin (H&E) staining, Safranin O/Fast Green staining, immunohistochemical staining for MMP13, terminal deoxynucleotidyl transferase-mediated dUTP nick-end labeling (TUNEL) staining, and Immunofluorescence (IF).

Under a stereomicroscope, articular cartilage was carefully isolated from the tibial plateau and femoral condyles of the left knee joints in each group. These cartilage samples were immediately snap-frozen in liquid nitrogen for subsequent RNA and protein extraction, which was used for the polymerase chain reaction (PCR) and Western blot analysis of MMP13.

Similarly, synovial membrane tissues were meticulously dissected from the left knee joint capsule and immediately snap-frozen in liquid nitrogen for the PCR and Western blot (WB) analysis of the components related to the Protein S-TAM-Rac1 axis.

### 2.6 Histological and histomorphometric analysis

H&E and Safranin O/Fast Green Staining: Paraffin-embedded knee joints were sectioned (5 μm) and stained. Cartilage damage was evaluated using the modified Mankin scoring system (structure, cells, matrix staining, tidemark integrity; 0–14 scale) (25) and the Osteoarthritis Research Society International (OARSI) grading system (0–6 scale) (26) by two blinded, independent observers. Synovitis was assessed using the Krenn synovitis score (synovial lining cell hyperplasia, stromal cellular density, inflammatory infiltrate; 0–9 scale) (27).

Immunohistochemistry (IHC): Paraffin sections (5 μm) of whole knee joints were incubated with anti-MMP13 primary antibody overnight at 4 °C. Immunoreactivity was visualized using DAB chromogen, followed by counterstaining with hematoxylin. The percentage of positively stained cells in cartilage and synovial regions was quantified in high-power fields per sample using ImageJ software.

TUNEL Staining: TUNEL Staining: Apoptotic cells in whole knee joint sections were detected using a TUNEL assay kit according to the manufacturer’s instructions, with DAPI used for nuclear counterstaining. TUNEL-positive (red) cells in the synovial region were counted and presented as a percentage of total DAPI-stained nuclei.

Immunofluorescence (IF) Staining: After deparaffinization to water and appropriate antigen retrieval, paraffin sections of the whole knee joint were blocked with 5% bovine serum albumin (BSA) at room temperature for 30 minutes. Subsequently, the sections were incubated overnight at 4 °C with a primary antibody mixture containing Cleaved Caspase-3 (an apoptotic executive protein) and F4/80 (a macrophage marker). Following rewarming, the corresponding fluorescently labeled secondary antibody mixture was added and incubated at room temperature for 1 hour in the dark. After that, the sections were stained with DAPI for nuclear counterstaining for 5 minutes and then mounted with anti-fluorescence quenching medium. Images were captured using a fluorescence microscope, with the focus on the synovial region. The apoptosis of macrophages in the synovial region was specifically observed by evaluating the double-labeled co-localization.

### 2.7 Enzyme-linked immunosorbent assay

Levels of IL-1β, TNF-α, IL-10, and TGF-β1 in serum were determined using commercial ELISA kits following the manufacturer’s instructions. Absorbance was read at 450 nm, and concentrations were calculated from standard curves.

### 2.8 Quantitative real-time polymerase chain reaction

Total RNA was extracted from tissues using TRIzol reagent. RNA concentration and purity were determined by spectrophotometry at 260 and 280 nm. Complementary DNA was synthesized from total RNA using a reverse transcription kit. RT−qPCR was performed in 10 μL reactions using the SYBR Green method, with each sample run in triplicate. Primers were designed using Primer 5 software and commercially synthesized. Relative gene expression was normalized to β−actin and calculated using the 2^^–ΔΔCt^ method. Primer sequences are provided in **Table 1**.

**Table 1:** Primer sequences used for qRT-PCR.

Primer name	Forward	Reverse	Product length
β-actin	ACATCCGTAAAGACCTCTATGCC	TACTCCTGCTTGCTGATCCAC	223bp
Axl	ACCGTGCCCGAAAGTCCTACA	GCCCTCCATCACAGCACCAAAT	167 bp
Rac1	TGCCTGCTCATCAGTTACACG	GGACGCAGTCTGTCATAATCTTC	155 bp
Mertk	TTCTGAACGAATCCAGCAACAA	CCTTTAGTGATGACGGCGATT	223bp
Pros1	TTGCCTTGTCCTTGGTGG	GTTCCGAGCACAGAGTTATGG	108bp

### 2.9 Western blot analysis

Synovial and cartilage tissues were separately homogenized in RIPA lysis buffer. Protein concentrations were determined using a BCA assay. Equal amounts of protein were separated by SDS−PAGE, transferred onto PVDF membranes, blocked, and incubated overnight with primary antibodies. After incubation with HRP−conjugated secondary antibodies, protein bands were visualized with an ECL substrate. Band intensities were quantified using ImageJ software and normalized to β−actin.

### 2.10 Statistical analysis

Data are presented as the mean ± standard deviation (Mean ± SD). Statistical analysis was performed using SPSS 26.0 software. The Shapiro−Wilk test was used to assess normality, and Levene’s test was applied to evaluate the homogeneity of variance. For data with a normal distribution and homogeneous variance, one−way analysis of variance (ANOVA) was performed, followed by Tukey’s multiple-comparison *post hoc* test. For data with heterogeneous variance, Welch’s ANOVA was used, followed by the Games-Howell *post hoc* test. A two-sided P<0.05 was considered statistically significant.

## 3 Results

### 3.1 EA ameliorates cartilage degradation in KOA rats

**Figure 2:**
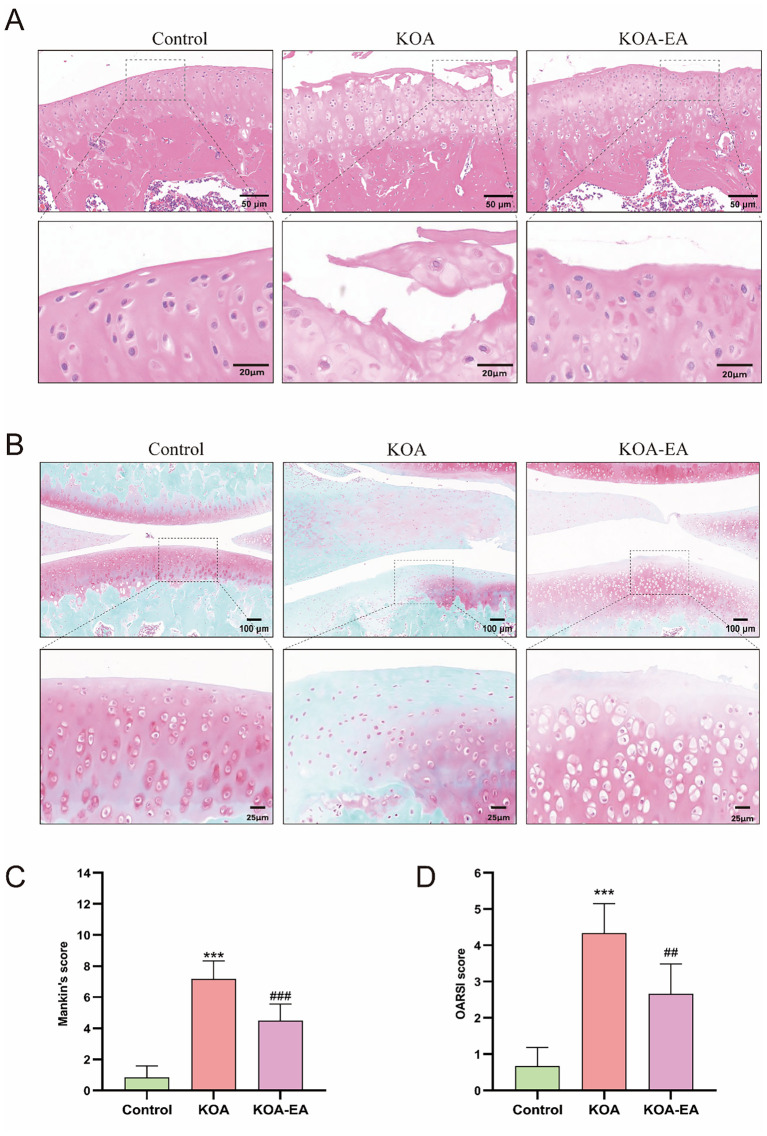
Cartilage histopathology. **(A)** Representative H&E images in articular cartilage. **(B)** Representative Safranin O/Fast Green staining images in articular cartilage. **(C)** Mankin’s scores. **(D)** OARSI scores. H&E-stained sections were imaged at original magnification ×20, with scale bars of 50 μm and 25 μm. Safranin O/Fast Green-stained sections were imaged at original magnification ×5, with scale bars of 100 μm and 25 μm. Data are presented as mean ± SD. n = 6 rats per group. ***P < 0.001 vs. Control; ^###^*P* < 0.001, ^##^*P* < 0.01 vs. KOA.

Histological assessment confirmed the successful induction of KOA. H&E staining revealed that cartilage in the Control group was intact with a smooth surface, evenly distributed chondrocytes, and clear tidal lines. In stark contrast, the KOA group exhibited severe surface irregularities, deep fissures, marked chondrocyte loss and cloning, and a disorganized structure. The KOA-EA group showed a notable improvement, with a smoother surface, reduced fissuring, more orderly chondrocyte arrangement, and a clearer tidal line compared to the KOA group (**Figure 2A**). Quantitative Mankin’s scores were significantly higher in the KOA group than in the Control group (*P* < 0.001). Compared with the KOA group, EA treatment significantly reduced the Mankin score of the KOA−EA group (*P* < 0.001) (**Figure 2C**).

Safranin O/Fast Green staining vividly depicted proteoglycan content. Control cartilage showed intense, uniform red staining. The KOA group displayed severe proteoglycan loss, evidenced by faint and uneven red staining, and a blurred or duplicated tidal mark. The KOA-EA group demonstrated moderate restoration of proteoglycan staining, with more homogeneous red color and a clearer tidal line (**Figure 2B**). Correspondingly, the OARSI score was markedly elevated in the KOA group versus Control (*P* < 0.001) and was significantly lowered by EA treatment (*P* < 0.01 vs. KOA group) (**Figure 2D**).

### 3.2 EA suppresses cartilage catabolism by downregulating MMP13

**Figure 3:**
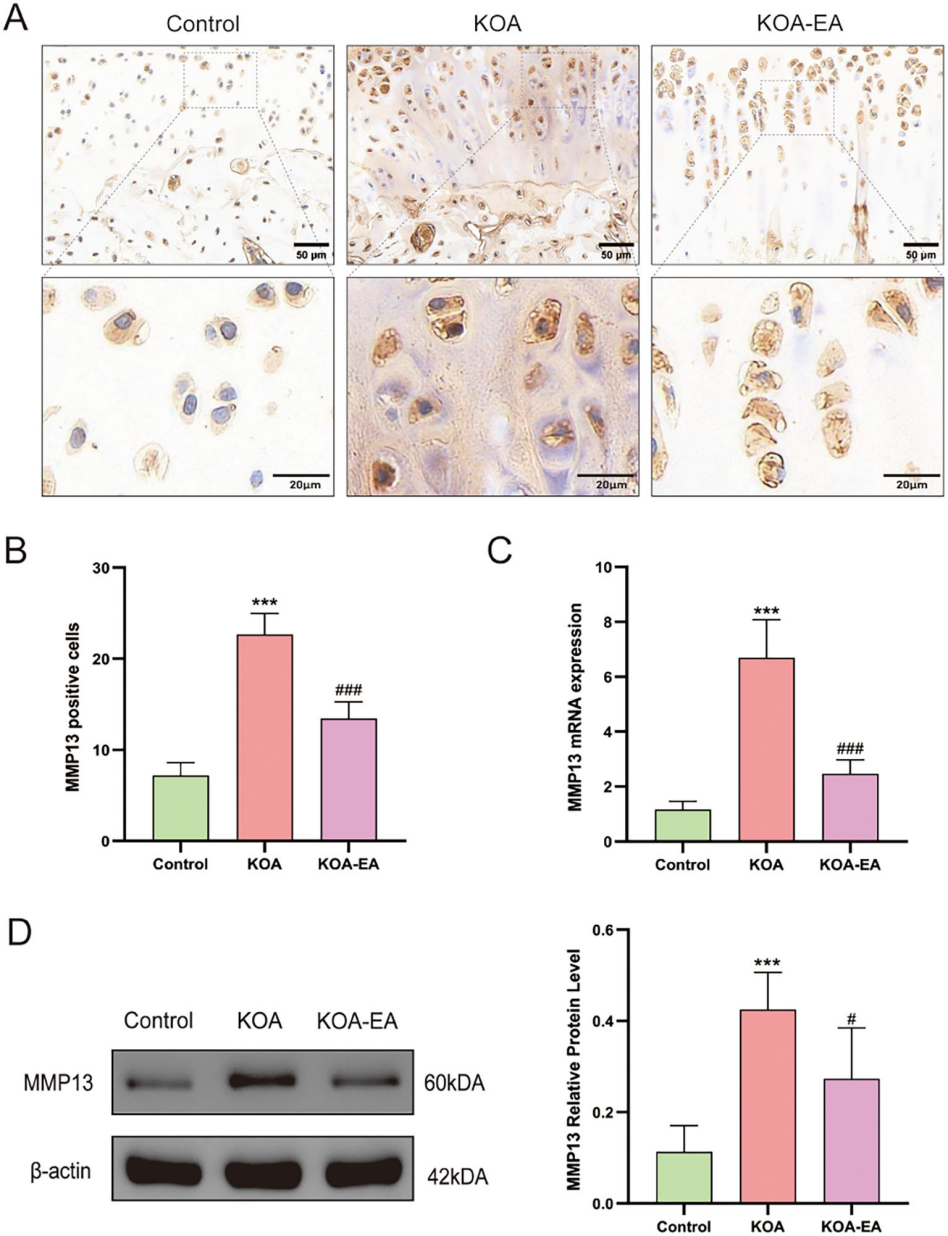
MMP13 expression in cartilage. **(A)** Representative immunohistochemical staining for MMP13 in articular cartilage. **(B)** Quantitative analysis of MMP13 immunostaining in articular cartilage. **(C)** Relative MMP13 mRNA expression in articular cartilage. **(D)** Representative western blot bands and densitometric analysis of MMP13 protein expression in articular cartilage. Immunohistochemical sections were imaged at original magnification ×20, with scale bars of 50 μm and 20 μm. MMP13 immunostaining was quantified using ImageJ by calculating the percentage of MMP13-positive cells in histological sections. n = 6 rats per group. Data are presented as mean ± SD. n = 6 rats per group. ***P < 0.001 vs. Control; ^###^*P* < 0.001, ^##^*P* < 0.01 vs. KOA.).

MMP13, a key collagenase in OA, was investigated. IHC showed minimal MMP13 expression in Control cartilage, which was intensely upregulated in the KOA group. EA treatment substantially reduced MMP13 immunoreactivity (**Figure 3A**). Quantitative analysis confirmed a significant increase in MMP13-positive cells in the KOA group (*P* < 0.001 vs. Control) and a significant decrease in the KOA-EA group (*P* < 0.001 vs. KOA) (**Figure 3B**). Consistently, qPCR and WB analysis of cartilage extracts revealed that both MMP13 mRNA and protein levels were dramatically elevated in the KOA model (*P* < 0.001 vs. Control) and were significantly suppressed by EA intervention (*P* < 0.001 for mRNA, *P* < 0.05 for protein vs. KOA) (**Figures 3C, D**).

### 3.3 EA attenuates synovial inflammation and apoptosis

**Figure 4:**
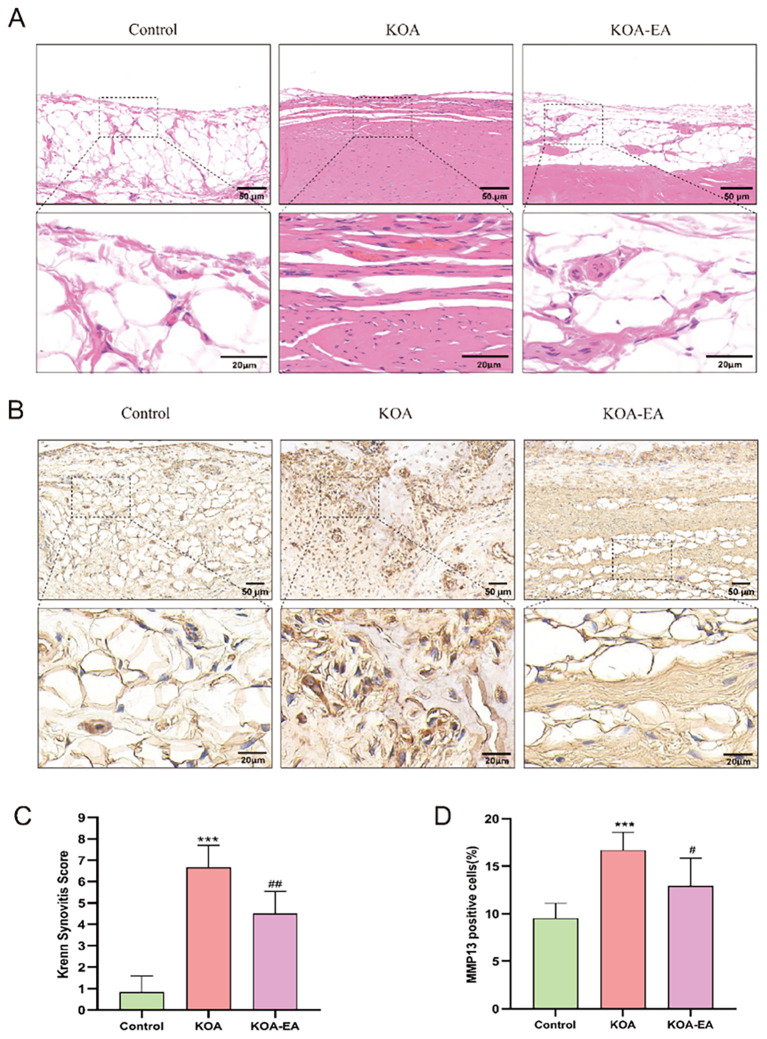
Synovial histopathology. **(A)** Representative H&E images in synovium. **(B)** Representative immunohistochemical staining for MMP13 in synovium. **(C)** Krenn scores. **(D)** Quantitative analysis of MMP13 immunostaining in synovium. Sections were imaged at original magnification ×20, with scale bars of 50 μm and 20 μm. MMP13 immunostaining was quantified using ImageJ by calculating the percentage of MMP13-positive cells in histological sections. Data are presented as mean ± SD. n = 6 rats per group. ***P < 0.001 vs. Control; ^##^*P* < 0.01, ^#^*P* < 0.05 vs. KOA.).

Synovial pathology was a primary focus. H&E staining of synovium revealed a thin lining layer and dense stroma with scant inflammatory cells in Controls. The KOA group exhibited pronounced synovial lining hyperplasia, dense stromal cellularity, and massive infiltration of mononuclear inflammatory cells. EA treatment effectively reduced synovial hyperplasia and inflammatory cell infiltration (**Figure 4A**). The Krenn synovitis score quantitatively mirrored these changes, being significantly higher in the KOA group than in Controls (*P* < 0.001) and significantly reduced by EA (*P* < 0.01 vs. KOA) (**Figure 4C**).

IHC for MMP13 in synovium showed a similar pattern: strong upregulation in KOA (*P* < 0.001 vs. Control) and significant downregulation after EA (*P* < 0.05 vs. KOA) (**Figures 4B, D**).

### 3.4 EA reduces synovial apoptosis and macrophage-associated apoptotic staining in a rat model of knee osteoarthritis

**Figure 5:**
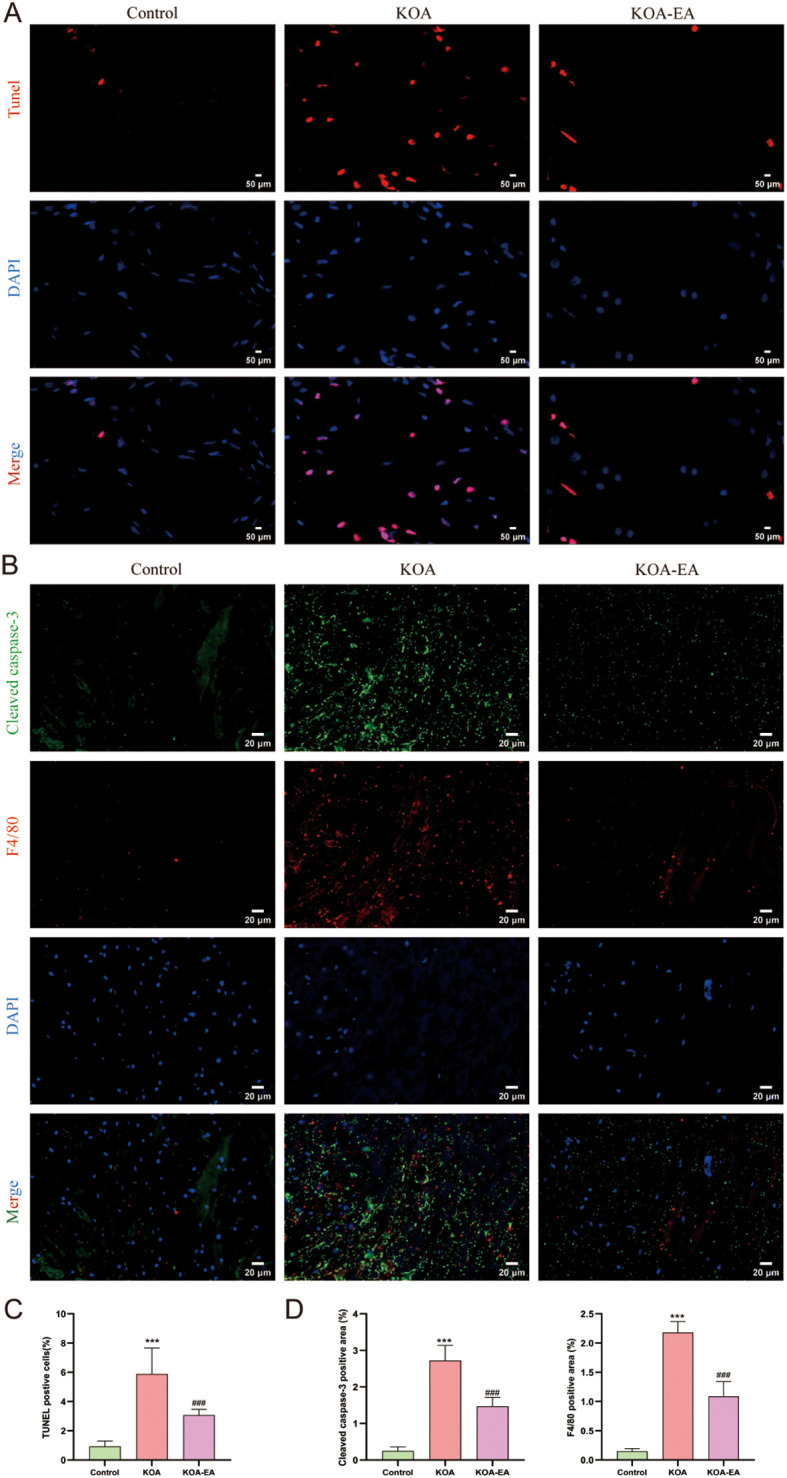
Apoptosis of synovial tissue macrophages. **(A)** Representative TUNEL staining of synovial tissue. **(B)** Representative immunofluorescence images showing the co-localization of cleaved caspase-3 (green) and F4/80 (red) in synovial tissue; nuclei were counterstained with DAPI (blue). **(C)** Quantification of TUNEL-positive cells in synovial tissue. **(D)** Quantitative analysis of the positive area fractions of active caspase-3 and F4/80 in synovial tissue. TUNEL-stained sections were imaged at original magnification ×40, with a scale bar of 50 μm. Immunofluorescence images were acquired at original magnification ×40, with a scale bar of 20 μm. The percentage of TUNEL-positive cells and the positive area fractions of active caspase-3 and F4/80 in tissue sections were quantified using ImageJ. Data are presented as mean ± SD. n = 6 rats per group. ***P < 0.001 vs. Control; ^###^*P* < 0.001 vs. KOA.

TUNEL staining revealed widespread apoptosis (red fluorescence) in the KOA synovium, which was markedly reduced in the KOA-EA group. The percentage of TUNEL-positive cells was significantly increased in the KOA group compared to Control group (*P* < 0.001) and was significantly decreased by EA treatment (*P* < 0.001 vs. KOA) (**Figures 5A, C**). Immunofluorescence double staining for Cleaved Caspase-3 (apoptosis executor) and F4/80 showed that some apoptosis-related signals in KOA synovium were associated with macrophages, and this co-localization was visibly reduced in the EA group. Compared with the control group, the KOA model group exhibited significantly higher positive area fractions of F4/80 and Cleaved caspase-3 (*P* < 0.001). Electroacupuncture treatment markedly reduced the positive area fractions of both F4/80 and cleaved caspase-3 compared with those in the KOA group (*P* < 0.001) (**Figures 5B, D**).

### 3.5 EA modulates systemic inflammatory and anti-inflammatory cytokines

**Figure 6:**
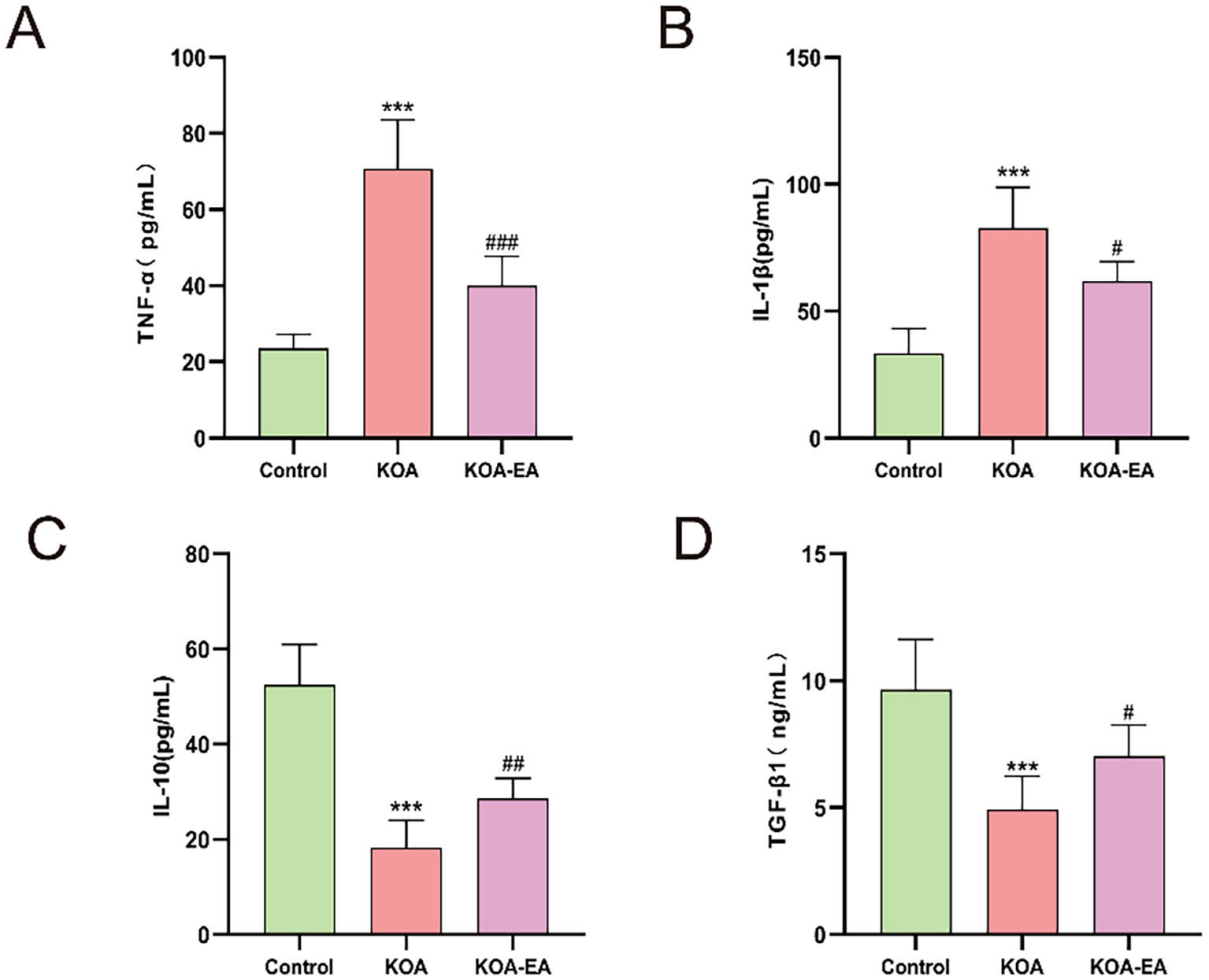
Serum cytokine profiles. **(A)** TNF-α. **(B)** IL-1β. **(C)** IL-10. **(D)** TGF-β1. Data are presented as mean ± SD. n = 8 rats per group. ***P < 0.001 vs. Control; ^###^*P* < 0.001, ^##^*P* < 0.01, ^#^*P* < 0.05 vs. KOA.

ELISA of serum revealed a profound systemic inflammatory state in KOA rats. Levels of the pro-inflammatory cytokines TNF-α and IL-1β were significantly elevated in the KOA group compared to Controls (*P* < 0.001 for both vs. KOA). EA treatment significantly reduced their levels (*P* < 0.001 for TNF-α, *P* < 0.05 for IL-1β) (**Figures 6A, B**). Conversely, levels of the anti-inflammatory and pro-resolving cytokines IL-10 and TGF-β1 were significantly suppressed in the KOA model (*P* < 0.001 for both). EA intervention effectively restored their levels, showing significant increases compared to the KOA group (*P* < 0.01 for IL-10, *P* < 0.05 for TGF-β1) (**Figures 6C, D**).

### 3.6 EA upregulates synovial mRNA expression of protein S–TAM–Rac1 axis-related genes

**Figure 7:**
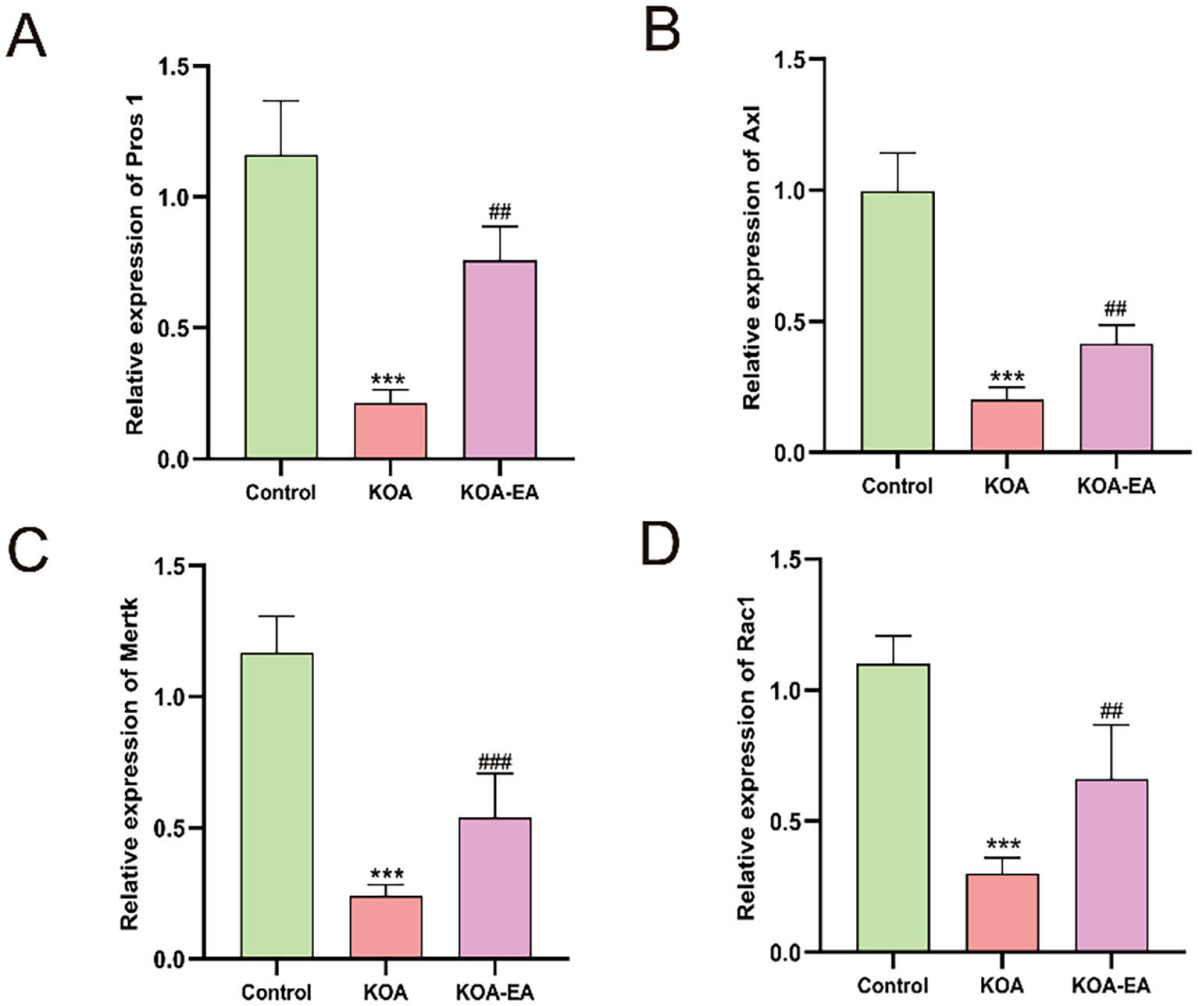
mRNA expression of Protein S–TAM–Rac1 axis-related genes in synovium (qRT-PCR). **(A)** Pros1. **(B)** Axl. **(C)** Mertk. **(D)** Rac1. Data are presented as mean ± SD. n = 6 rats per group. ***P < 0.001 vs. Control; ^###^*P* < 0.001, ^##^*P* < 0.01 vs. KOA.

qRT-PCR analysis of synovial tissue showed marked downregulation of key molecules in the Protein S-TAM-Rac1 axis axis in KOA. The mRNA expression levels of Pros1, Axl, Mertk, and Rac1 were all significantly decreased in the KOA group compared with the Control group (*P* < 0.001 for all) (**Figures 7A–D**). EA treatment was associated with reversal of this downregulation, leading to significant increases in all four transcripts compared with the untreated KOA group (Pros1, Axl, Rac1: *P* < 0.01; Mertk: *P* < 0.001).

### 3.7 EA is associated with partial restoration of synovial protein expression of protein S–TAM–Rac1 axis-related molecules

**Figure 8:**
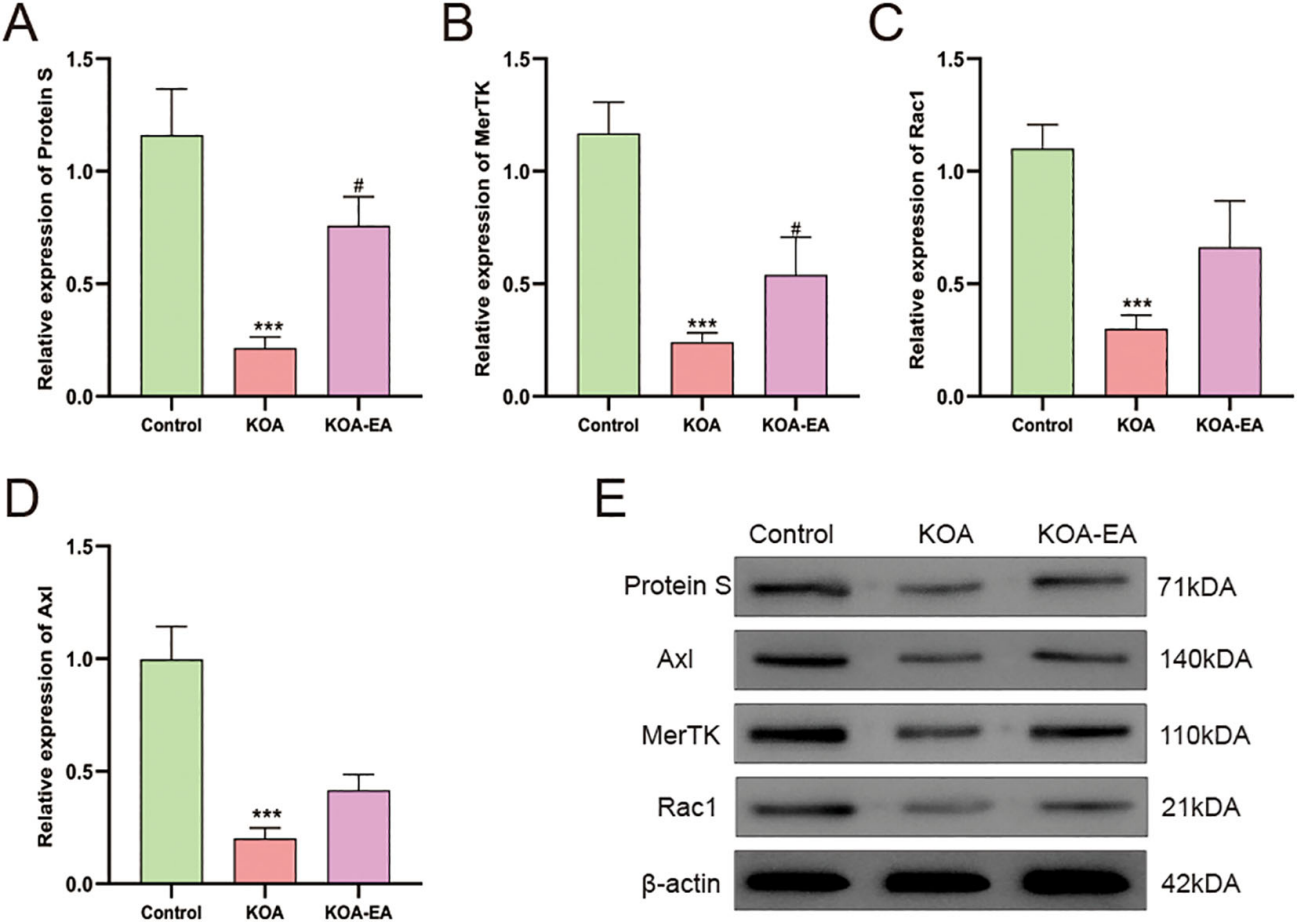
Protein expression of the Protein S-TAM-Rac1 axis in synovium determined by Western blot. **(A)** Representative Western blot bands. **(B-E)** Densitometric analysis of Protein S, Axl, MerTK, and Rac1 protein levels normalized to GAPDH. Data are presented as mean ± SD. n = 6 rats per group. ***P < 0.001 vs. Control; ^#^*P* < 0.05 vs. KOA.

Western blot analysis corroborated the PCR findings at the protein level. Representative blots (**Figure 8E**) and densitometric quantification (**Figures 8A–D**) demonstrated that the protein expression levels of Protein S, Axl, MerTK, and Rac1 were substantially lower in KOA synovium than in the Controls (*P* < 0.001 for all). EA treatment was associated with increased expression of these proteins. Compared with the KOA group, Protein S and MerTK were significantly upregulated (*P* < 0.05), whereas Axl and Rac1 exhibited an upward trend that did not reach statistical significance.

## 4 Discussion

This study provides evidence that long-term EA intervention improved both structural joint damage and synovial inflammation in a rat model of post-traumatic KOA. The demonstrated findings include improved cartilage histology, reduced synovial hyperplasia and inflammatory infiltration, lower apoptosis-related staining, decreased MMP13 expression, a shift in serum cytokines toward a less inflammatory profile, and increased synovial mRNA expression of Pros1, Axl, Mertk, and Rac1, together with significantly increased protein expression of Protein S and MerTK and upward trends in Axl and Rac1 after EA.

The transition of KOA from a “cartilage-centric” to a “whole-joint” disease model has brought synovitis to the forefront (28). Our ACLT model reproduced synovial hyperplasia, inflammatory infiltration, and elevated Krenn scores alongside cartilage degeneration. The inflamed synovium is an important source of catabolic mediators such as IL-1β, TNF-α, and MMP13, which can contribute to cartilage damage and pain (29). In the present study, the reductions in synovial MMP13 and serum IL-1β/TNF-α after EA were accompanied by lower Mankin’s and OARSI scores. This pattern is consistent with clinical observations linking synovitis severity with pain and structural progression (30). These findings suggest that synovitis may be an important therapeutic target in KOA.

Efferocytosis is a major component of inflammation resolution (16, 31). Among the regulatory pathways involved, the TAM receptor system—especially Axl and MerTK—has been recognized as a major mediator of efferocytosis in macrophages. Binding of Protein S to these receptors is thought to elicit two critical cellular responses: Rac1-dependent cytoskeletal rearrangement that supports the phagocytic “eat-me” signal, as well as an anti-inflammatory program that inhibits NF-κB activity and promotes the expression of TGF-β1 and IL-10 (32–34). In our study, the mRNA expression of Pros1, Axl, Mertk, and Rac1 and the protein expression of Protein S, Axl, MerTK, and Rac1 were all downregulated in KOA synovium. EA treatment significantly increased the mRNA expression of these molecules, while at the protein level, Protein S and MerTK were significantly upregulated and Axl and Rac1 showed upward trends. In parallel, KOA synovium showed increased TUNEL positivity and Cleaved Caspase-3/F4/80 co-localization. These changes were attenuated following electroacupuncture intervention. These findings suggest an accumulation of apoptotic cells in the synovial tissue of KOA rats, with defective efferocytosis as a possible indirect contributor. Apoptotic bodies that are not efficiently cleared may undergo secondary necrosis and release damage-associated molecular patterns (DAMPs), thereby further aggravating local inflammation and contributing to a self-perpetuating vicious cycle (18). A similar pattern of impaired inflammatory resolution has been reported in rheumatoid arthritis (35). Accordingly, the improvement in KOA synovitis following electroacupuncture may be associated with changes in macrophage efferocytosis-related processes.

The observed changes in Protein S, Axl, MerTK, and Rac1 may be relevant to the phenotypic improvements seen after EA treatment, but this interpretation requires further direct investigation. Similarly, the reduction in apoptotic cell accumulation and the attenuation of synovitis may be related to changes in macrophage efferocytosis-related pathways. In addition, the altered cytokine profile may also be relevant, as TGF-β1 and IL-10 have been linked to TAM receptor signaling and inflammatory resolution (21). Overall, the present findings support an association between EA treatment and changes in synovial inflammatory status, while the underlying mechanisms remain to be further clarified.

Although the present whole-synovium analyses do not allow definitive cell-type assignment, the most plausible EA-responsive populations are synovial macrophages and fibroblast-like synoviocytes. This interpretation is supported by the established role of Axl and MerTK in macrophage-mediated inflammatory resolution and by emerging evidence that OA synovial pathology is shaped by macrophage-fibroblast crosstalk, with Axl signaling also detectable in osteoarthritic FLS and synovial explants (22, 29, 36). Accordingly, EA may preferentially enhance a macrophage-centered pro-resolving program while secondarily modulating FLS inflammatory activity; however, this remains inferential and requires cell-specific validation.

The possibility that intra-articular Protein S alone might reproduce part of the therapeutic effect is mechanistically plausible but should be interpreted cautiously. Local Protein S supplementation could, in principle, augment TAM-dependent pro-resolving signaling if synovial Axl/MerTK receptor competence is preserved (33, 36). However, EA is likely to exert broader neuroimmune and tissue-regulatory effects that extend beyond a single ligand-receptor pathway (37–39). Protein S should therefore be viewed as a candidate partial mediator rather than an assumed substitute for EA, pending direct comparative experiments.

Angiogenesis is biologically relevant to synovitis; however, it was not directly evaluated in the present ACLT model, as markers such as CD31, VEGF, α-SMA, and microvessel density were not assessed. Therefore, we cannot determine from the current data whether EA exerts anti-angiogenic effects in KOA. This question should be addressed in future studies, especially given recent evidence that acupuncture-related interventions may modulate angiogenesis-associated changes in experimental KOA (26).

These findings also support the view that EA may be most useful as part of a multimodal treatment strategy. Rational combinations for future study may include EA with exercise-based rehabilitation, weight-management programs in metabolically at-risk patients, or selected intra-articular therapies targeting the inflammatory joint microenvironment. Recent evidence suggests that acupuncture combined with active exercise training may improve pain and function beyond either intervention alone, and that electroacupuncture may represent one of the more effective acupuncture modalities for KOA symptom control (13, 40). Whether such combinations can also yield additive anti-synovitic or structure-modifying effects remains an important translational question.

The durability of EA after treatment cessation was not evaluated in the present study, because all endpoints were collected immediately after the intervention period. We therefore cannot determine whether the observed benefits would persist or gradually diminish after withdrawal. Although clinical evidence suggests that acupuncture-related effects in KOA may be sustained for a period after treatment in some settings (41), this cannot be directly extrapolated to the ACLT rat model. Future studies incorporating post-treatment washout intervals will be necessary to define the persistence of the structural, inflammatory, and molecular changes observed here.

How peripheral stimulation at distant acupoints is associated with these molecular changes within the joint remains unclear. Our study identifies the molecular and phenotypic changes associated with EA treatment, but the upstream mechanisms were not directly investigated. One possible explanation involves neuroimmune communication, as EA has been reported to activate sensory afferent nerves that transmit signals to the spinal cord and brainstem (37). On this basis, downstream efferent pathways, including the cholinergic anti-inflammatory pathway and the sympatho-adrenal axis, may be relevant to the observed anti-inflammatory effects (38, 39, 42). These pathways have been implicated in the regulation of macrophage function and cytokine production and may therefore contribute to changes in the synovial microenvironment. In addition, systemic mediators induced by EA, such as endogenous opioids or glucocorticoids, may also be involved (37). However, these possibilities remain speculative in the context of the present study and require direct experimental validation.

Several limitations should be acknowledged. First, only male rats were included, which may limit generalizability because OA progression and immune responses can differ by sex. Second, sham-EA and non-acupoint stimulation controls were not included, which limits conclusions regarding acupoint specificity and non-specific electrical stimulation effects. Third, the sample size for tissue-based analyses was relatively limited. Finally, although EA treatment was associated with changes in the Protein S-TAM-Rac1 axis, direct functional validation of this pathway and direct assessment of efferocytosis were not performed. Future studies incorporating sham-EA controls, direct efferocytosis assays, pharmacological or genetic pathway blockade, and both male and female animals are needed.

## 5 Conclusion

In conclusion, EA alleviated synovitis and cartilage degeneration in an experimental KOA model and was associated with upregulation of synovial Protein S–TAM–Rac1 axis-related molecules, with stronger evidence at the mRNA level than at the protein level. The accompanying reduction in apoptosis-related staining and inflammatory mediators suggests that EA may influence inflammation-resolution pathways. However, because direct efferocytosis assays and pathway-interference experiments were not performed, the mechanistic involvement of the Protein S-TAM (Axl/MerTK)-Rac1 axis should be interpreted as provisional.

## Data Availability

The original contributions presented in the study are included in the article/**Supplementary Material**. Further inquiries can be directed to the corresponding author.
