# Supplementary figures and images for: Electroacupuncture attenuates synovitis in knee osteoarthritis and is associated with modulation of the protein S-TAM (Axl/MerTK)-Rac1 signaling axis
Source: Front Immunol. 2026 Jul 9;17:1815290. doi: 10.3389/fimmu.2026.1815290 (PMC13391395; doi:10.3389/fimmu.2026.1815290)

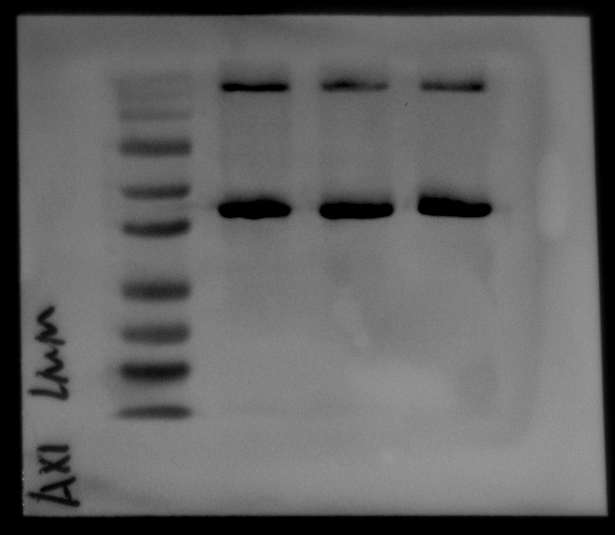

Supplement: Supplementary file 2 [file Image1.tif]

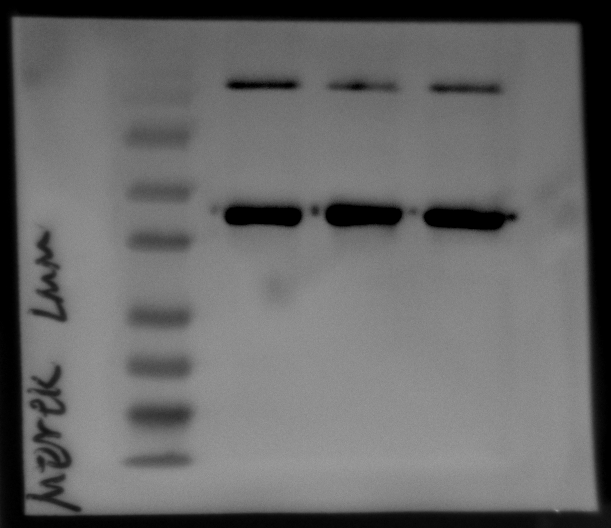

Supplement: Supplementary file 3 [file Image2.tif]

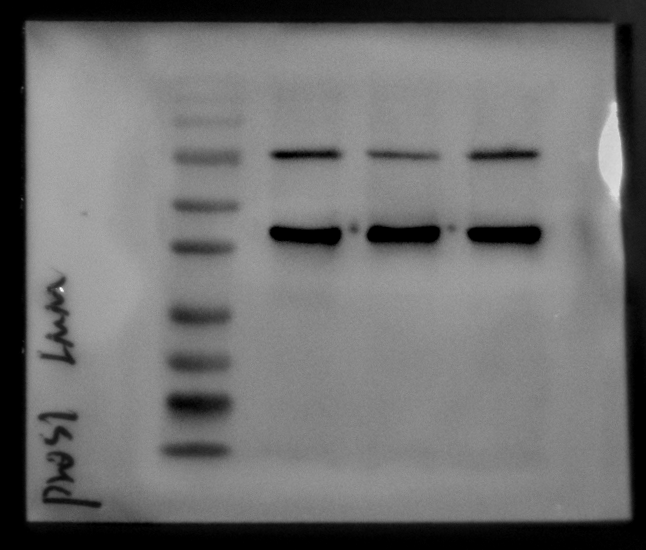

Supplement: Supplementary file 4 [file Image3.tif]

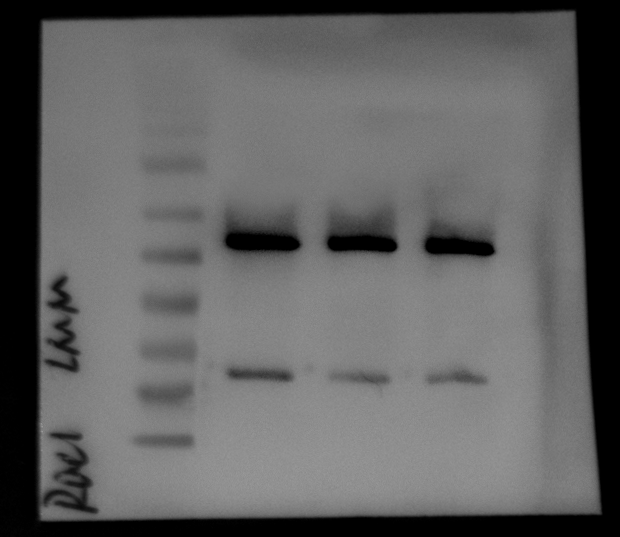

Supplement: Supplementary file 5 [file Image4.tif]

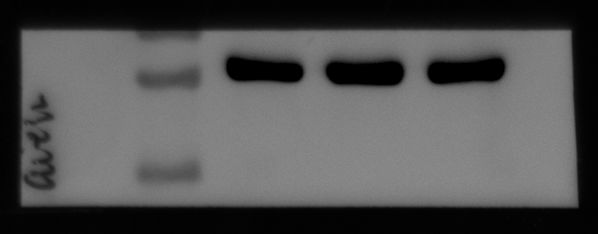

Supplement: Supplementary file 6 [file Image5.tif]

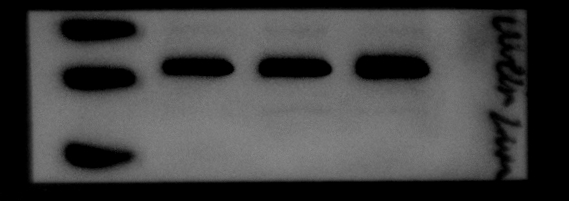

Supplement: Supplementary file 7 [file Image6.tif]
